# Patient reported outcomes of MDD patients treated with esketamine nasal spray in real world practice: the French ELLIPSE study

**DOI:** 10.1192/j.eurpsy.2026.10194

**Published:** 2026-07-31

**Authors:** P.-M. Llorca, L. Samalin, E. Olié, I. Rodriguez, J. Dupin, C. Wicart, P. Demaricourt

**Affiliations:** 1 Department of Psychiatry, CHU Clermont-Ferrand, University of Clermont Auvergne, CNRS, Clermont Auvergne INP, Institute Pascal (UMR 6602), Clermont-Ferrand; 2 Psychiatric Emergency and Post Emergency Department, Pole Urgence, Centre Hospitalier Universitaire de Montpellier, Hôpital Lapeyronie, Montpellier; 3 Medical Affairs, Johnson&Johnson, Issy-les-Moulineaux; 4 Psychiatry and Neurosciences, Sainte Anne Hospital GHU Paris, Paris, France

## Abstract

**Introduction:**

Esketamine nasal spray (ESK) was approved in 2019 in Europe for adults with Treatment-Resistant Depression (TRD), defined as inadequate response to at least two different antidepressants during the current moderate to severe depressive episode

**Objectives:**

To assess patient-reported outcomes related to symptomatology, quality of life, and occupational impairment over 12 months following ESK initiation in patients with Major Depressive Disorder (MDD)

**Methods:**

ELLIPSE is a French, prospective, multicenter, non-interventional study describing MDD patients treated with ESK. Patient-reported outcomes were collected using QIDS-SR16, QLDS, and WPAI:D questionnaires throughout the 12-month follow-up (Baseline, D8, M1, M2, M3, M6, M9, M12).

**Results:**

A total of 200 MDD patients (including 195 [97.5%] treatment resistant patients) were enrolled across 31 sites.

Among them, 108 (54.0%) were employed at baseline, including 79 on sick leave. The remaining 92 patients had no occupation at baseline, and 50 (25.0%) cited the current depressive episode as the reason, while 30 (15.0%) were retired.

QIDS response rates (improvement from baseline ≥ 50% or score ≤ 5) in patients treated with ESK increased to 37.7% at Month 2 (M2) and 45.0% at Month 3 (M3), then plateaued (Figure 1). QLDS response (improvement from baseline ≥ 8, (Rozjabek et al. 2022)) followed a similar trend, reaching 26.5% at M2 in patients undertreatment, with sustained response over the follow-up in both the total population and the patients still under treatment (Figure 2). WPAI:D scores showed consistent improvement across all four domains in patients still under treatment at the visit (Figure 3).

**Image 1:**
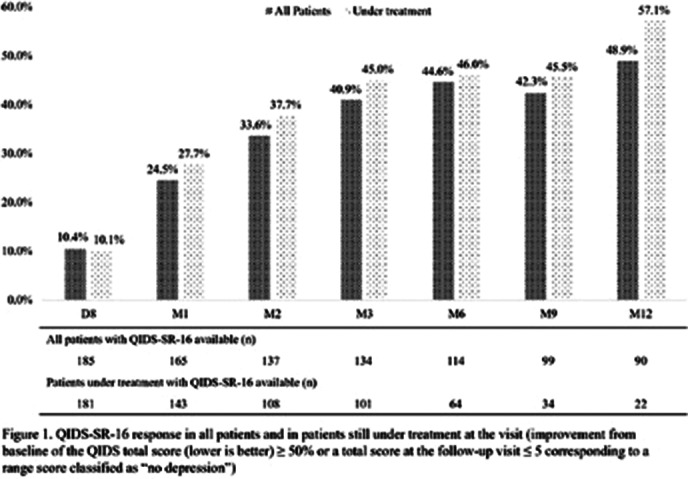
Image 1: Long description.

**Image 2:**
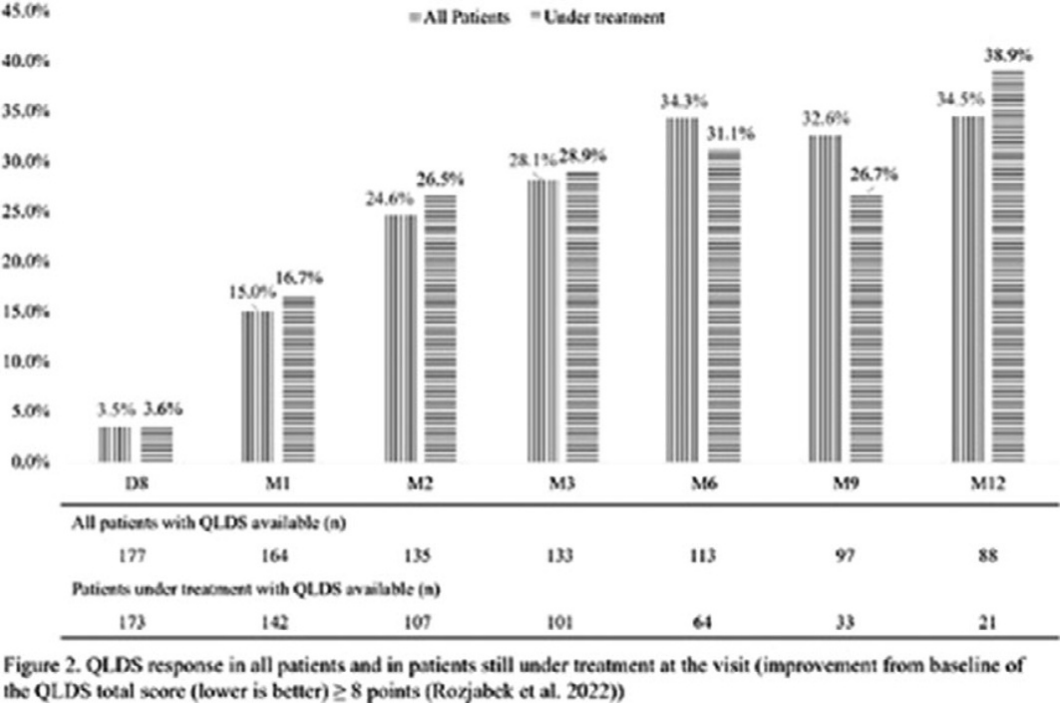
Image 2: Long description.

**Image 3:**
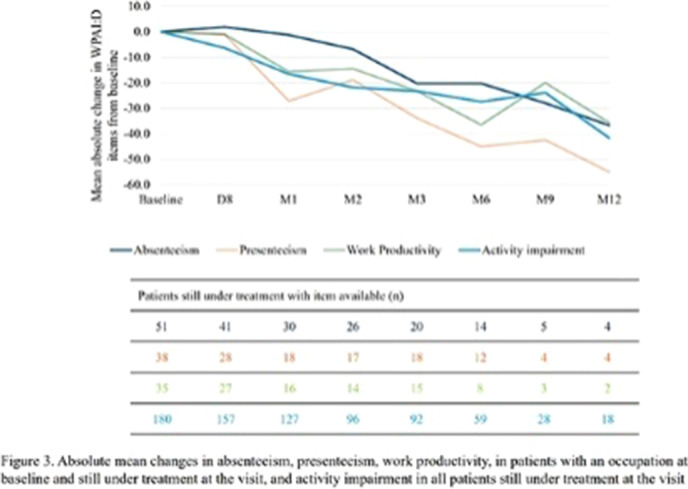
Image 3: Long description.

**Conclusions:**

ELLIPSE is the first large-scale, real-world study in France to evaluate esketamine in MDD, offering a 360-degree view of the patient evaluation. By integrating patient-reported outcomes on depressive symptoms, quality of life, and occupational functioning, the study highlights the central role of the patient perspective in assessing treatment effectiveness. These findings reinforce the relevance of ESK in the therapeutic arsenal for TRD.

**Disclosure of Interest:**

P.-M. Llorca Consultant of: Johnson&Johnson consultant, L. Samalin Consultant of: Johnson&Johnson consultant, E. Olié Consultant of: Johnson&Johnson consultant, I. Rodriguez Employee of: Johnson&Johnson employee, J. Dupin Employee of: Johnson&Johnson employee, C. Wicart Employee of: Johnson&Johnson employee, P. Demaricourt Consultant of: Johnson&Johnson consultant

## Long descriptions

### Image 1: Long description

A grouped bar chart and data table. The y-axis represents percentage response from 0.0 percent to 60.0 percent. The x-axis shows time points: D 8, M 1, M 2, M 3, M 6, M 9, and M 12.

Legend:

* Solid grey bars: All Patients.

* Dotted bars: Under treatment.

Data Trends:

* D 8: All Patients 10.4 percent, Under treatment 10.1 percent.

* M 1: All Patients 24.5 percent, Under treatment 27.7 percent.

* M 2: All Patients 33.6 percent, Under treatment 37.7 percent.

* M 3: All Patients 40.9 percent, Under treatment 45.0 percent.

* M 6: All Patients 44.6 percent, Under treatment 46.0 percent.

* M 9: All Patients 42.3 percent, Under treatment 45.5 percent.

* M 12: All Patients 48.9 percent, Under treatment 57.1 percent.

Data Table below the x-axis (n values):

* All patients with Q I D S dash S R dash 16 available: 185, 165, 137, 134, 114, 99, 90.

* Patients under treatment with Q I D S dash S R dash 16 available: 181, 143, 108, 101, 64, 34, 22.

Navigate back to Image 1:.

### Image 2: Long description

A bar chart with a Y-axis representing percentage from 0.0% to 45.0% and an X-axis representing time points. The legend at the top indicates vertical striped bars for All Patients and horizontal striped bars for Under treatment.

Data points from left to right:

* D 8: All Patients 3.5%, Under treatment 3.6%.

* M 1: All Patients 15.0%, Under treatment 16.7%.

* M 2: All Patients 24.6%, Under treatment 26.5%.

* M 3: All Patients 28.1%, Under treatment 28.9%.

* M 6: All Patients 34.3%, Under treatment 31.1%.

* M 9: All Patients 32.6%, Under treatment 26.7%.

* M 12: All Patients 34.5%, Under treatment 38.9%.

Below the chart is a data table for sample sizes (n):

* All patients with Q L D S available (n): 177 at D 8, 164 at M 1, 135 at M 2, 133 at M 3, 113 at M 6, 97 at M 9, and 88 at M 12.

* Patients under treatment with Q L D S available (n): 173 at D 8, 142 at M 1, 107 at M 2, 101 at M 3, 64 at M 6, 33 at M 9, and 21 at M 12.

Navigate back to Image 2:.

### Image 3: Long description

A line graph is positioned at the top. The Y-axis is labeled Mean absolute change in W P A I D items from baseline, with a scale from 10.0 down to negative 60.0. The X-axis represents time points: Baseline, D 8, M 1, M 2, M 3, M 6, M 9, and M 12.

Four data series are plotted, all showing a general downward trend (improvement) over time:

* Absenteeism (dark blue line): Starts at 0, remains highest, ending near negative 35 at M 12.

* Presenteeism (orange line): Shows the sharpest decline, reaching approximately negative 55 at M 12.

* Work Productivity (green line): Fluctuates, ending near negative 35 at M 12.

* Activity impairment (light blue line): Shows a steady decline to approximately negative 42 at M 12.

Below the graph is a legend corresponding to the line colors.

At the bottom is a table titled Patients still under treatment with item available (n). It contains four rows of numbers color-coded to match the graph lines, showing the sample size (n) decreasing over time from Baseline to M 12:

* Row 1 (Dark Blue): 51, 41, 30, 26, 20, 14, 5, 4.

* Row 2 (Orange): 38, 28, 18, 17, 18, 12, 4, 4.

* Row 3 (Green): 35, 27, 16, 14, 15, 8, 3, 2.

* Row 4 (Light Blue): 180, 157, 127, 96, 92, 59, 28, 18.

Navigate back to Image 3:.

